# An oleaginous endophyte *Bacillus subtilis* HB1310 isolated from thin-shelled walnut and its utilization of cotton stalk hydrolysate for lipid production

**DOI:** 10.1186/s13068-014-0152-4

**Published:** 2014-10-14

**Authors:** Qin Zhang, Yanbin Li, Liming Xia

**Affiliations:** Key Laboratory of Biomass Chemical Engineering of Ministry of Education, Department of Chemical and Biological Engineering, Zhejiang University, Hangzhou, 310027 China; College of Life Science, Tarim University, Alaer, 843300 Xinjiang China; Xinjiang Production & Construction Corps Key Laboratory of Protection and Utilization of Biological Resources in Tarim Basin, Tarim University, Alaer, 843300 Xinjiang China

**Keywords:** Oleaginous endophyte, *Bacillus subtilis* HB1310, Thin-shelled walnut, Cotton stalk hydrolysate, Lipid production

## Abstract

**Background:**

Third generation biodiesel processing from microbial lipids using low-cost lignocellulosic feedstocks has attracted much attention. Endophytes isolated from oleaginous plants possibly have the capacity to accumulate lipids similar to the hosts. However, little work has been reported in terms of endophytic bacteria isolation from oleaginous plants and their lipid production using lignocellulosic hydrolysate as substrate.

**Results:**

A new oleaginous endophyte HB1310 has been isolated from the thin-shelled walnut, and identified as *Bacillus subtilis* on the basis of both 16S rDNA gene sequencing and examination of its physiological and biochemical properties. This strain effectively accumulates cellular lipids using cotton stalk hydrolysate as a substrate. The optimum C/N ratio, culture temperature, and pH value were determined to be 50/1, 30°C, and 6.5, respectively. Batch fermentation was conducted in a bioreactor using these parameters. Satisfactory production, with a maximum lipid productivity of 2.3 g/L, lipid content of 39.8% (w/w), and cell dry weight (CDW) of 5.7 g/L, was obtained at a culture time of 48 h. Variations in the fluorescent intensity and lipid inclusion formation of cells collected at different sampling times illustrate the potential of this bacterium to be useful for cellular lipid production. The fatty acid profile of the produced bacterial lipids showed that the major constituents are myristic, palmitic, stearic, oleic, and linoleic acids with an estimated cetane number of about 61.8, indicating that this strain may be suitable for biodiesel production.

**Conclusions:**

The present investigation is the first report of an oleaginous endophytic bacterium isolated from the thin-shelled walnut. This strain is capable of producing high lipid contents rapidly using cotton stalk hydrolysate as a substrate, and its lipids are suitable for use as the feedstock for biodiesel production.

## Background

Biodiesel is considered to be a promising alternative to traditional hydrocarbon fuels, as it can be produced from a variety of sources using techniques such as chemical conversion of triglycerides into fatty acyl methyl esters. In recent years, thirdgeneration methods of biodiesel processing based on microbial lipids have been a common subject of research [1-3]. Microbial lipids (also called single cell oils, SCOs) are accumulated in oleaginous microorganisms, which are defined as microbes that can accommodate lipid contents of more than 20% of their dry biomass [4,5]. Yeasts and algae are the most studied oleaginous microorganisms at the moment due to their high cellular lipid contents. In contrast to these organisms, only a few bacteria are capable of accumulating a significant amount of lipids [6,7]; most bacteria produce lipids only on the plasma membrane, resulting in a lipid content of less than 20% of dry weight. Certain bacteria native to special environments may be more prone to produce cellular lipids; for example, the *Rhodococcus opacus* strain PD630 was isolated from a soil sample collected near a gas-works plant in Germany, and can accumulate high bacterial lipid contents using hydrocarbons as a substrate [8]. Two bacterial associates of marine sponges, *Bacillus subtilis* (RRL-8) and *Pseudomonas* spp. (RRL-28), were found to be capable of producing total lipids of 33.4 and 42.7% respectively, with a C/N ratio of 50 [9].

In recent years, endophytes have been the subject of significant attention worldwide, as it has been found that they play an important role in biological control and promotion of plant growth as well as in inducing many bioactive metabolites [10-12]. Moreover, many endophytic fungi isolated from oleaginous plants have been reported to have the capacity to accumulate high lipid contents [13,14]. Dey *et al.* [15] isolated two endophytic fungi, *Colletotrichum* sp. and *Alternaria* sp., from *Ocimum sanctum* and *Brassica juncea*, respectively, which were found to be potential candidates for use as biodiesel feedstocks [15]. However, few endophytic bacteria have been isolated from oleaginous plants for the purpose of microbial lipid production. In particular, there have been no reports of oleaginous microbes isolated from the thin-shelled walnut, a nutrient-rich nut planted widely in Xinjiang, China. The thin-shelled walnut has been reported to contain lipid contents of more than 60% of its dry fruit weight [16], and thus lipid-rich tissues constitute the endogenous environment of this species. As a result, it is likely possible to be able to isolate oleaginous microbes from the thin-shelled walnut.

Interest in biofuel production from lignocellulosic feedstocks has increased during the early years of the twenty-first century. Achieving bioconversion of xylose, the second most abundant sugar present in lignocellulosic biomass after glucose, is crucial for the development of cost-effective bioprocesses for fuel production [17]. Some oleaginous yeasts are able to utilize both the glucose and xylose found in lignocellulosic hydrolysate for lipid production [18-20]. However, few oleaginous bacteria can metabolize xylose. For example, *R. opacus* PD630 was only able to utilize the xylose in lignocellulosic hydrolysate once its xylose metabolism had been genetically engineered [17]. Isolation of oleaginous bacteria which can metabolize xylose inherently is vital for producing lipids from lignocellulosic hydrolysate.

Cotton stalks, which are generated as by-products from cotton cultivation in large quantities (6 to 8 million tons per year in Xinjiang, China alone), may be useful lignocellulosic feedstocks. In recent years, high-value utilization of cotton stalks has been of interest, especially as fermentation of cotton stalk hydrolysate can produce high-value chemicals like bioethanol and xylitol [21,22]. However, there are no reports concerning the fermentation of cotton stalk hydrolysate for lipid production using oleaginous microbes. As such, this study was undertaken with the following goals: i) isolation and identification of an oleaginous endophyte from the native variety of the thin-shelled walnut, *Akesu* 185, which is widely cultivated in Xinjiang, China; ii) analysis of the process of lipid production using cotton stalk hydrolysate as a substrate to determine the optimum fermentation conditions for maximum cellular lipid production; iii) analysis of the fatty acid profile of the resulting endophytic isolate, and evaluation of its potential use as a biodiesel feedstock.

## Results and discussion

### Isolation and identification of endophyte from thin-shelled walnut

The mature fruit of the thin-shelled walnut contains a high content of walnut oil. Several endophytic bacteria inhabit this high-oil-content endogenous environment. Six bacteria were isolated using the LB, WA, and DI1 media. All six bacteria were then inoculated on a DI2 plate for further isolation. It was found that only one bacterium, designated as number HB1310, was able to grow well on the DI2 plate. This strain was also inoculated on a control plate containing a solution identical to DI2 but lacking the cotton stalk hydrolysate. This strain grew very little and demonstrated no single colony formation on the control plate after culturing 48 h, while single colonies with diameters of 3to 5 mm were produced when the strain was streaked several times on a DI2 plate. Similarly, the bacterium could produce single colonies on a DI2 plate using the dilution-plate method with a 10^5^ dilution (the inoculation number was approximately 3.9 × 10^6^ cfu/mL) after culturing 48 h, whereas it could hardly produce a single colony at the same dilution on a control plate, indicating that the strain has a preference for using cotton stalk hydrolysate as its carbon source. As such, strain HB1310 was regarded as a preliminary candidate endophyte for utilization of the reducing sugars in cotton stalk hydrolysate. The bacterium has a rod shape with a size of approximately 0.8-1.2 μm × 1.5-4.0 μm. The Gram-staining result of this bacterium was positive, and endospores could be observed during certain growth periods. The endophyte HB1310 was found to have 99% sequence similarity with *Bacillus subtillis* [GenBank:HM027881.1] by testing against the available 16S rDNA gene sequences from the GenBank database. Moreover, the 16S rDNA gene sequence of strain HB1310 was submitted to GenBank by our group, and the accession number KJ636450 was obtained. The physiological and biochemical properties were also examined, and the results are summarized in Table 1. Taking into account the results of the 16S rDNA gene sequence matching as well as the physiological and biochemical properties of the bacterium, the endophyte HB1310 can be conclusively identified as *Bacillus subtillis*.

**Table 1:** **Physiological and biochemical properties of** ***Bacillus subtillis*** **HB1310**

**Items**	**Results**	**Items**	**Results**
Gram staining	+	Shape	Rod
Citrate utilization	+	Gelatin liquefaction	+
Methylred test	-	Production of indole	+
V-P test	+	Production of H_2_S	-
Starch hydrolysis	+	Litmus milk	-
Lipase	-	Urease	-

+: positive; -: negative.

This bacterium is able to utilize various carbon sources and nitrogen sources to fuel its growth, and this particular strain can also grow successfully over broad temperature and pH ranges (Table 2). Interestingly, when glucose and xylose were supplied as carbon sources and yeast extract was used as the nitrogen source, endophyte HB1310 was able to effectively accumulate cellular lipids, with the lipid content reaching levels of greater than 20% (w/w) (Table 2). When cultured at temperatures between 30 and 45°C and within a pH range of 6.5 to 7.0, the endophyte grew extremely well, with an OD_600_ of higher than 0.5, and accumulated cellular lipid content of more than 20% (w/w) (Table 2).

**Table 2:** **Growth and lipid production of** ***Bacillus subtillis*** **HB1310 with different carbon sources, nitrogen sources, temperatures, and pH values**

**Carbon source**	**Citrate**	**Glucose**	**Xylose**	**Sucrose**	**Mannitol**	**Sodium acetate**	**Lactose**	**Arabinose**
Growth	+	++	++	+	++	-	+	-
Lipid production	+	++	++	+	+	-	+	-
Nitrogen source	Ammonium sulfate	Yeast extract	Ammonium nitrate	Potassium nitrate	Sodium nitrate	Urea	Peptone	Ammonium bicarbonate
Growth	+	++	++	++	+	+	++	+
Lipid production	+	++	+	+	+	+	+	+
Culture temperature (°C)	4	20	30	37	45	55	65	75
Growth	+	+	++	++	++	+	+	+
Lipid production	-	+	++	++	++	+	+	+
Culture pH value	5.5	6.0	6.5	7.0	7.5	8.0	8.5	9.0
Growth	+	++	++	++	++	+	+	+
Lipid production	-	+	++	++	+	+	+	+

The carbon source and nitrogen source utilization experiments were developed using basal medium described in the *Identification Manual of Systematic Bacteriology*, with culture temperature 37°C and pH7.0. The temperature and pH experiments were developed using DI2 medium; in temperature experiments, the pH value was modulated to 7.0, and in pH experiments, the culture temperature was 37°C. In the row labeled Growth, “++” represented that the OD_600_ was higher than 0.5, “+” represented that 0.1 < OD_600_ < 0.5, and “-”represented that the OD_600_ was close to 0 with a culture time of 48 h. In the row labeled Lipid production, “++” represented that the lipid content was higher than 20% (w/w), “+” represented that the lipid content was lower than 20% (w/w), and “-”represented that the lipid could hardly be detected with a culture time of 48 h.

### The effects of C/N ratio on lipid production and cell dry weight (CDW)

**Figure 1:**
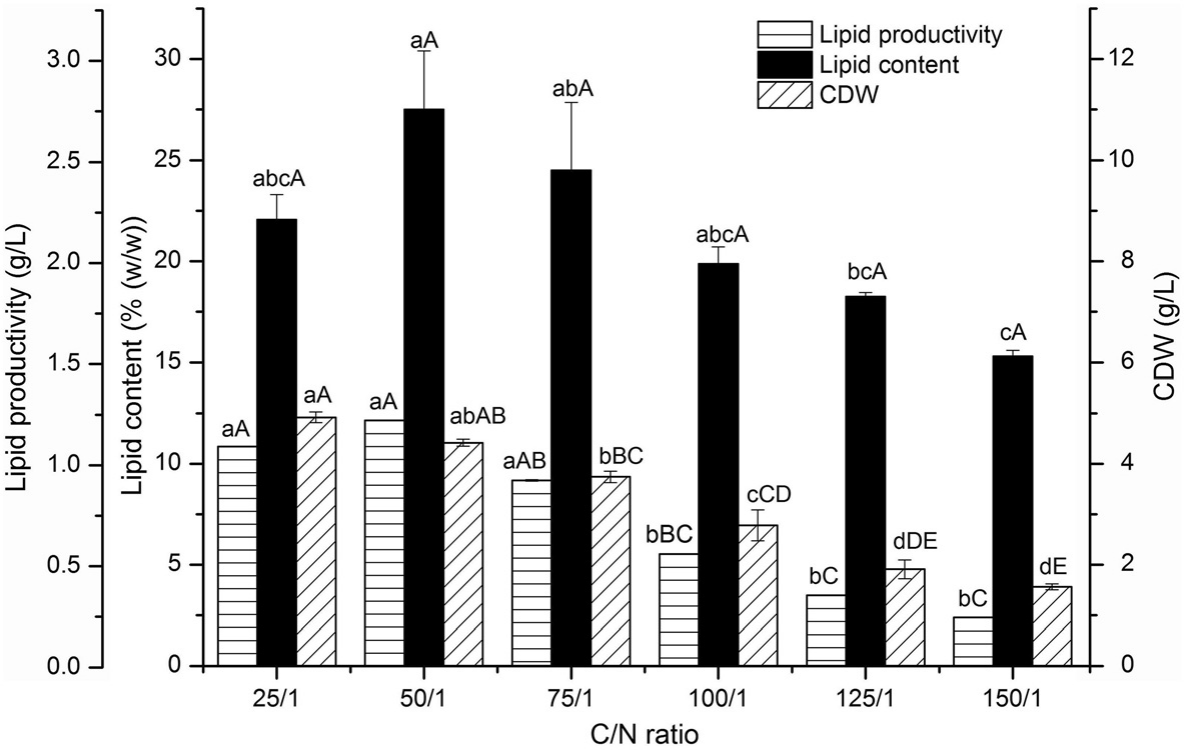
**Effects of C/N ratios on lipid productivity, lipid content, and CDW of endophyte HB1310.** The error bars represent the standard deviation of three independent replicates. The lowercase letters above the columns represent differences among the mean values significant at a level of 5% (*P* < 0.05), and the capital letters above the columns represent differences among the mean values highly significant at a level of 1% (*P* < 0.01) using the Tukeytest.

A preliminary screening of carbon and nitrogen source utilization patterns (Table 2) revealed that endophyte HB1310 tends to accumulate lipids when glucose and xylose are provided as the carbon sources, which indicates that this bacterium may be able to effectively utilize lignocellulosic hydrolysate. Yeast extract was shown to be the optimum nitrogen source. As such, cotton stalk hydrolysate (which has been shown to consist mainly of glucose and xylose in a 40:10 proportion [22], with trace amounts of other reducing sugars) was provided as the carbon source for fermentation experiments, and yeast extract was added as the nitrogen source. The C/N ratio was adjusted over a series of concentration gradients, from 25/1 to 150/1, by changing the ratio of hydrolytic reducing sugars to yeast extract. The initial concentration of reducing sugar was 50 g/L in all experiments, while varying C/N ratios were achieved by adding yeast extract. After culturing for 48 h, cells were collected and the accumulated cellular lipids were extracted and measured. The results show that the optimum lipid productivity and lipid content were obtained at a C/N ratio of 50/1, showing significant differences with those at other C/N ratios at a level of 5%. The highest cell dry weight (CDW) was obtained at a C/N ratio of 25/1 with high significance at levels of 5% and 1% (Figure 1). The amount of CDW present decreased sharply with increases in the C/N ratios. High C/N ratios also led to low lipid content, indicating that the accumulation of cellular lipids in endophyte HB1310 is not limited by nitrogen concentration, which is different from the lipid-accumulation behavior observed in many oleaginous yeasts and filamentous fungi [23-25]. However, the optimum C/N ratio was identical to that found for a bacterial associate of marine sponges, *Bacillus subtilis* (RRL-8), which was able to accumulate a maximum lipid content of 33.4% [9]. It may be concluded that a C/N ratio of approximately 50 creates the most beneficial conditions for high lipid content accumulation by *Bacillus subtilis* HB1310.

### The effects of temperature and pH value on lipid production, cell dry weight, and residual sugars

**Figure 2:**
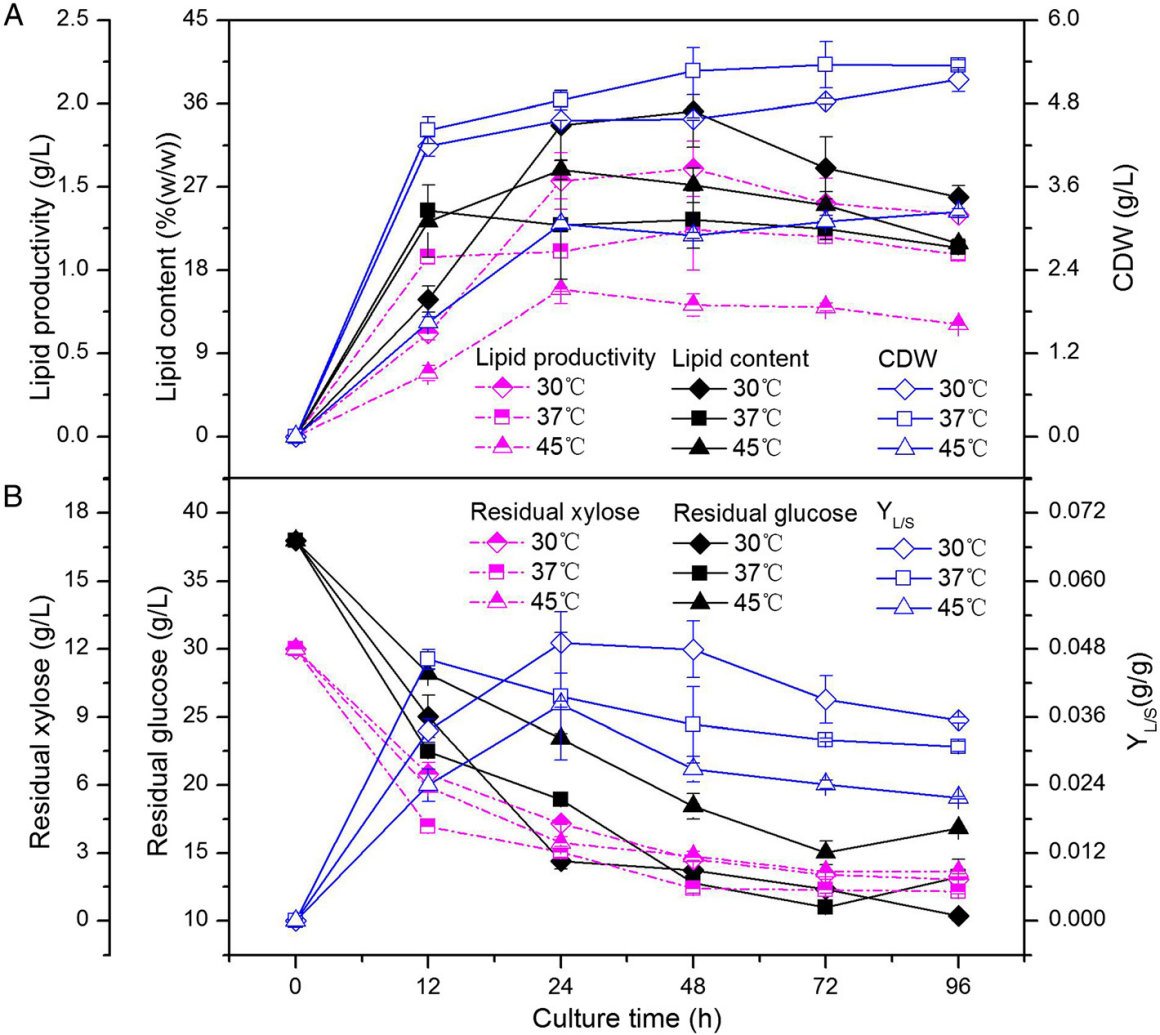
**Time course of lipid productivity, lipid content, CDW (A), and residual sugars, Y**_**L/S**_ **(B), of endophyte HB1310 at 30, 37, and 45°C.** The error bars represent the standard deviation of three independent replicates.

As it was identified as *Bacillus subtillis* in the results mentioned above, the endophyte HB1310 is a bacterium inherent in generating endospore, which enables this strain to grow over a wide temperature range (Table 2). As this strain can produce cellular lipids to loadings of more than 20% when cultured at 30, 37, or 45°C, the lipid productivity, lipid content, CDW, residual sugars, and Y_L/S_ were examined at each of these three temperatures. Endophyte HB1310 grew rapidly at all three culture temperatures. The maximum CDW produced was 5.4 g/L, which was obtained after culturing at 37°C for 72 h, while the maximum lipid content, 35.2% (w/w), was obtained after 48 h at 30°C (Figure 2A). The curves in Figure 2A show the evolution of CDW and lipid productivity and content over the culture time, with the highest CDW and lowest lipid productivity and content both produced at 37°C, while culturing at 30°C led to not only high CDW but also the highest lipid productivity and lipid content. The bacterium was also able to effectively utilize the glucose and xylose found in cotton stalk hydrolysate for cell growth and lipid accumulation, with continuously decreasing concentrations of reducing sugars over the first 48 h (Figure 2B). The concentrations of glucose and xylose decreased at least 50% and 75%, respectively, at all three temperatures after 48 h of culture time. From 48 to 96 h, the residual sugars stayed relatively stable, with the lowest residual sugars obtained at 37°C (Figure 2B). Moreover, considering the lipid yield produced from reducing sugars, the highest Y_L/S_ was obtained at 30°C, indicating that the bacterium gained the maximum conversion rate of reducing sugars to lipid when culturing at 30°C. Combined with the above analysis of lipid content and biomass, it may be that reducing sugar contributes mainly to cell biomass formation at temperatures between 30 and 37°C, while it effectively contributes to both cell biomass formation and lipid production at 30°C. In oleaginous microorganisms, lipid accumulation is generally believed to be a (partial) growth-coupled biochemical process, with the cell growth and lipid production stages not clearly distinguishable from one another [26]. In this study, 30°C was considered to be the optimum temperature for lipid production by endophyte HB1310 due to the higher biomass and highest lipid content produced at this temperature.

**Figure 3:**
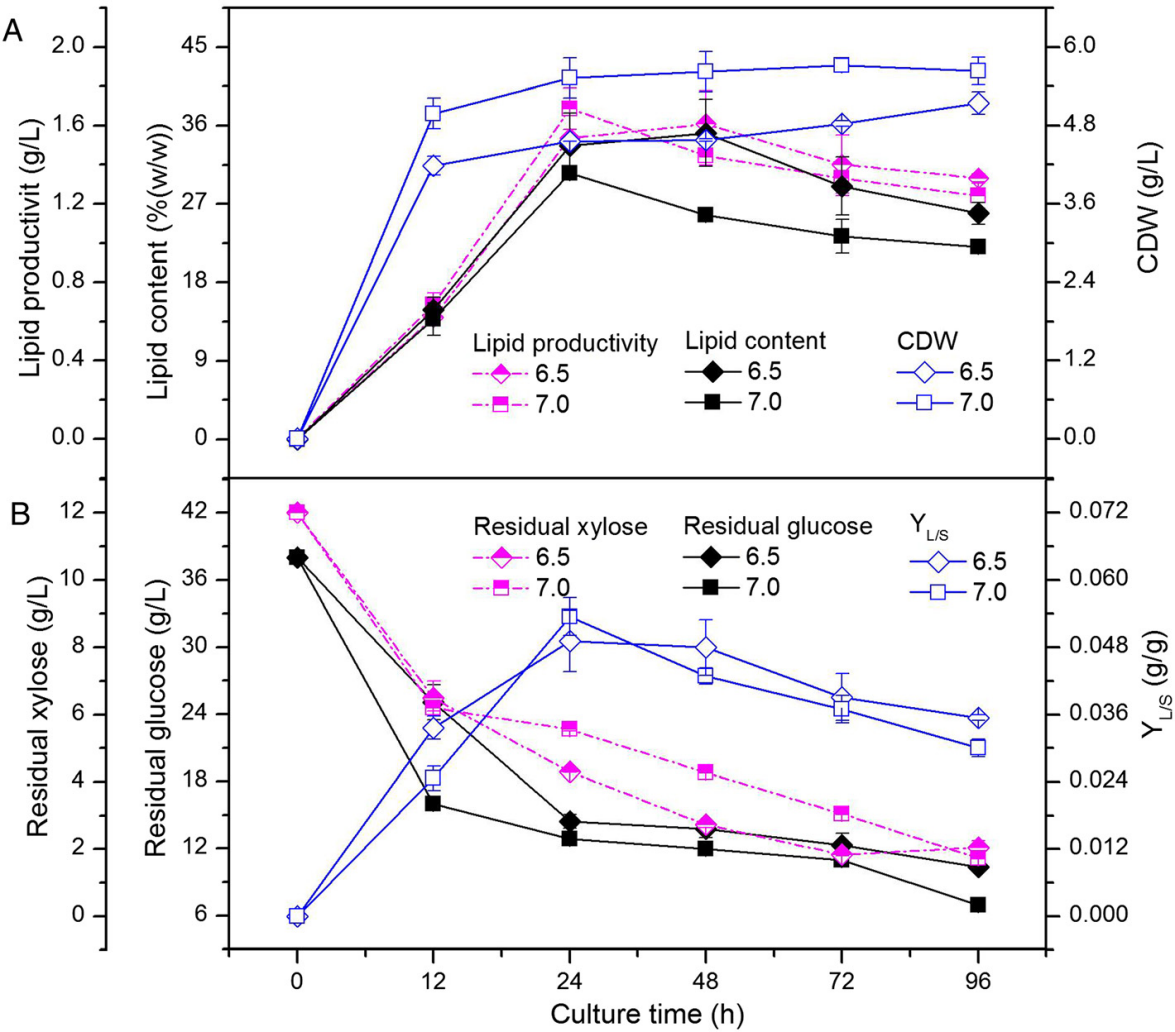
**Time course of lipid productivity, lipid content, CDW (A), and residual sugars, Y**_**L/S**_ **(B), of endophyte HB1310 at pH 6.5 and 7.0.** The error bars represent the standard deviation of three independent replicates.

The results in Table 2 show that while endophyte HB1310 is capable of growing over a broad pH range, lipid contents of greater than 20% (w/w) were achieved only in a relatively limited range near neutral pH, from 6.5 to 7.0. Therefore, the lipid productivity, lipid content, CDW, residual glucose, and residual xylose were also tested at pH 6.5 and 7.0. The maximum CDW was obtained at pH 7.0, while the maximum lipid productivity and content were obtained at pH 6.5 (Figure 3A). The residual glucose and xylose decreased continuously over the culture time, reaching values of lower than 13.8 and 2.8 g/L, respectively, after incubating for 48 h (Figure 3B). As both lower concentration of xylose and higher lipid productivity, lipid content, and Y_L/S_ were obtained at pH 6.5, this condition was regarded as the optimum pH value for lipid production.

### Analysis of lipid production process in bioreactor

**Figure 4:**
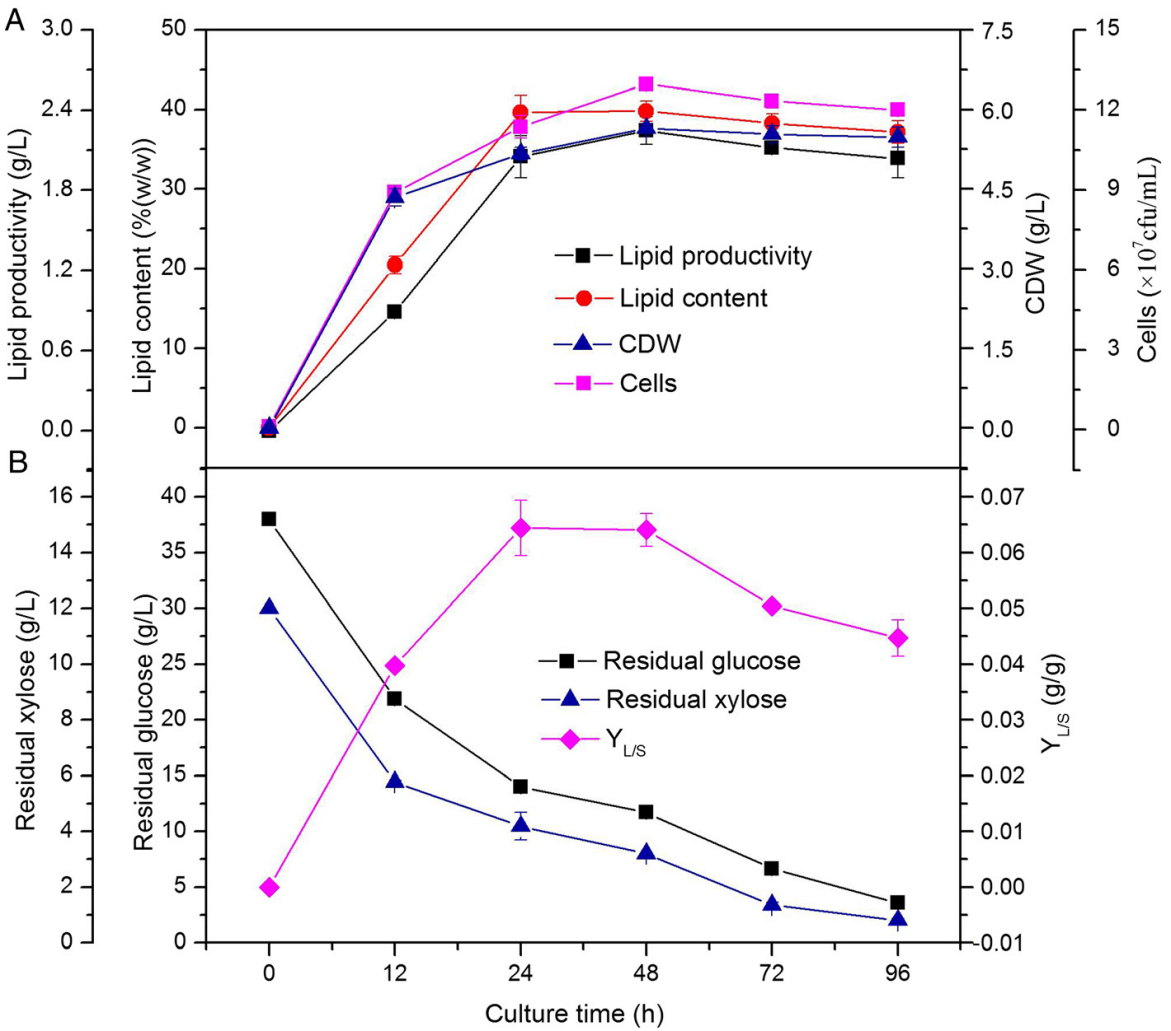
**Time course of lipid productivity, lipid content, CDW (A), and residual sugars, Y**_**L/S**_ **(B), of batch fermentation of endophyte HB1310 in bioreactor.** The error bars represent the standard deviation of three independent replicates.

Using the information from the above results, lipid production through fermentation with endophyte HB1310 was monitored in a 10-L bioreactor using cotton stalk hydrolysate as the substrate. The culture conditions were similar to those used in the experiments conducted in the 500-mL flasks, with the previouslydetermined optimum values of key factors as follows: C/N ratio of 50/1, culture temperature of 30°C, and pH 6.5. In this experiment, fermentation products were sampled at culture times of 12, 24, 48, 72, and 96 h. The lipid productivity, lipid content, CDW, residual glucose, residual xylose, Y_L/S_, and bacterial viable counts were analyzed for each time point. Batch fermentation in the 10-L bioreactor resulted in higher lipid production, cell growth rate, and sugar-consuming rate, compared with the shake-flask tests mentioned above. Impressive productivities were obtained using this method, with a maximum lipid productivity of 2.3 g/L, lipid content of 39.8% (w/w), and cell dry weight (CDW) of 5.7 g/L achieved at a culture time of 48 h (Figure 4A). The trend of the bacterial viable counts over the culture time also demonstrates that the bacterial cells were able to propagate rapidly, multiplying logarithmically between 0 and 12 h. The highest cell number, 13.0 × 10^7^ cfu/mL, was obtained at 48 h, after which the viable counts decreased somewhat (Figure 4A). This drop in viable counts might result from delayed germination of endospores, indicating the possibility that many endospores may be present at the later stages of fermentation. In addition, very effective utilization of glucose and xylose was demonstrated in this experiment. After 96 h of fermentation, the concentrations of glucose and xylose decreased about 90.6% and 93.2%, respectively, and the Y_L/S_ reached higher than 0.06 g/g (Figure 4B), implying that the reducing sugars in the fermentation medium were almost completely consumed by the endophyte for lipid production.

**Figure 5:**
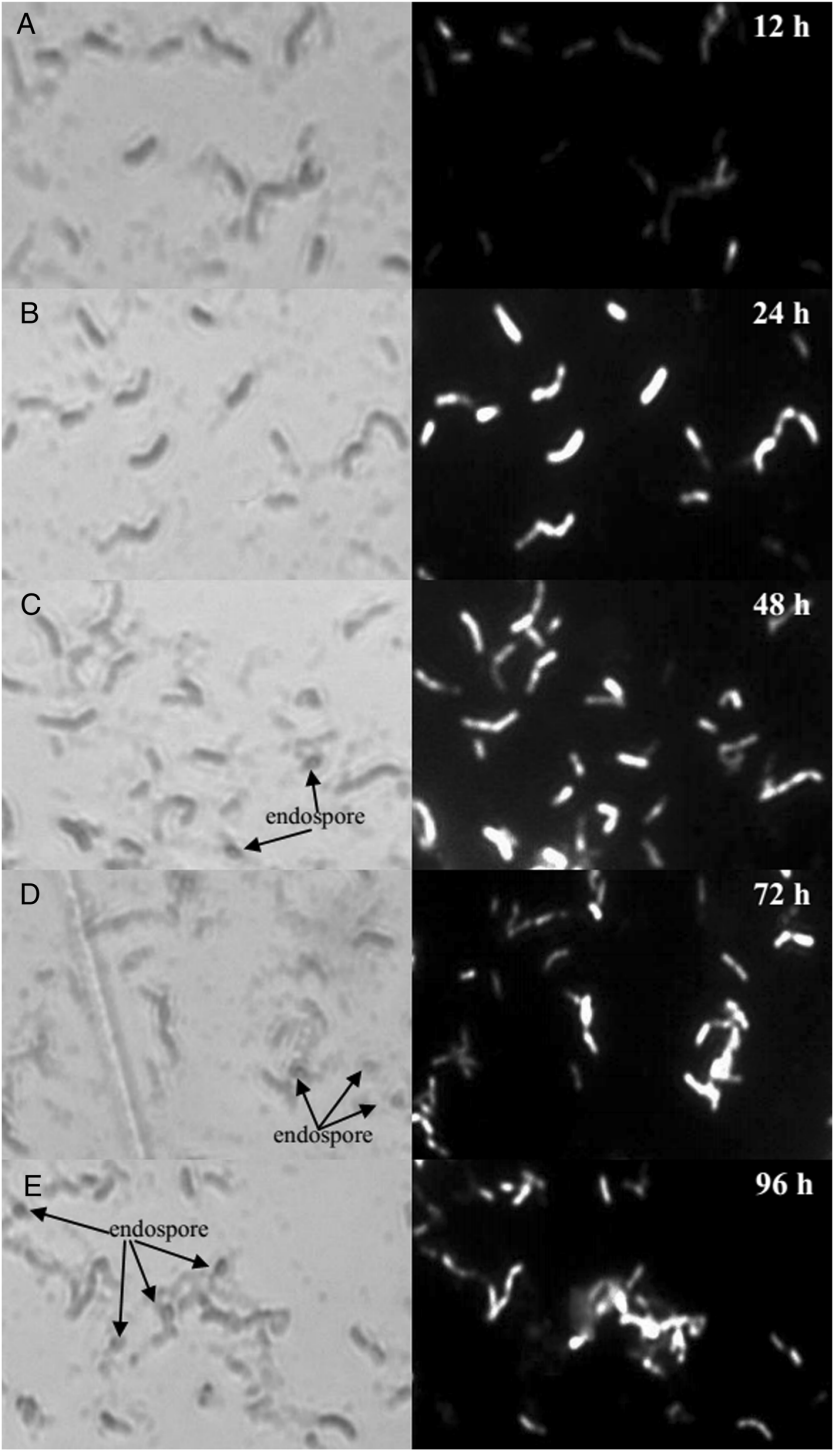
**Phase contrast of photographs of Nile red stained cells by light microscopy (left) and fluorescence microscopy (right) sampled at 12 (A), 24 (B), 48 (C), 72 (D), and 96 h (E).**

In order to enable further understanding of cellular lipid formation in endophyte HB1310, cells withdrawn at 12, 24, 48, 72, and 96 h were examined using fluorescence microscopy and transmission electron microscopy (TEM). Wältermann *et al.* [27] studied the mechanism of lipid-body biogenesis in *Acinetobacter calcoaceticus* ADP1 and *R. opacus* PD630 by applying the fluorescent dye Nile red, and found that prokaryotic neutral lipid accumulation is initiated at the cytoplasm membrane and that free cytoplasmic lipidbodies were only developed at a later stage [27]. In this study, the cells sampled at 12 h displayed weaker fluorescence than at other time points (Figure 5A), and some of this can be clearly observed to be localized at the peripheries of cells, indicating that these lipid inclusions might also be initiated at the cytoplasmic membrane. The fluorescent intensity of the cells increased after 24 h (Figure 5B-E), implying that mature lipid inclusions may have been formed at later stages of fermentation. Few endospores were observed in the sample taken at 24 h, as compared to the many endospores found in the samples taken from 48 to 96 h. These endospores were clearly visible using an optical microscope (Figure 5C-E). These observations suggest that there is a prolonged period (of at least 24 h) during which nutritive cell growth occurs without formation of endospores in a fermentation medium made with cotton stalk hydrolysate.

Endospore formation may influence lipid accumulation in bacterial cells. Areas with endospores fluoresce weakly or not at all, as can be seen by comparing the images taken with the optical and fluorescence microscopes (Figure 5C-E). This observation can be combined with the result (found in Figure 4A) that while the bacterial cells maintained almost constant levels of biomass, the lipid content declined from 72 to 96 h, which suggests that the massive endospores formed at later stages of fermentation caused a decrease in lipid content. Therefore, in order to maximize lipid production, the fermentation of cotton stalk hydrolysate by endophyte HB1310 should be terminated within 72 h, with 48 h being the optimal culture time.

**Figure 6:**
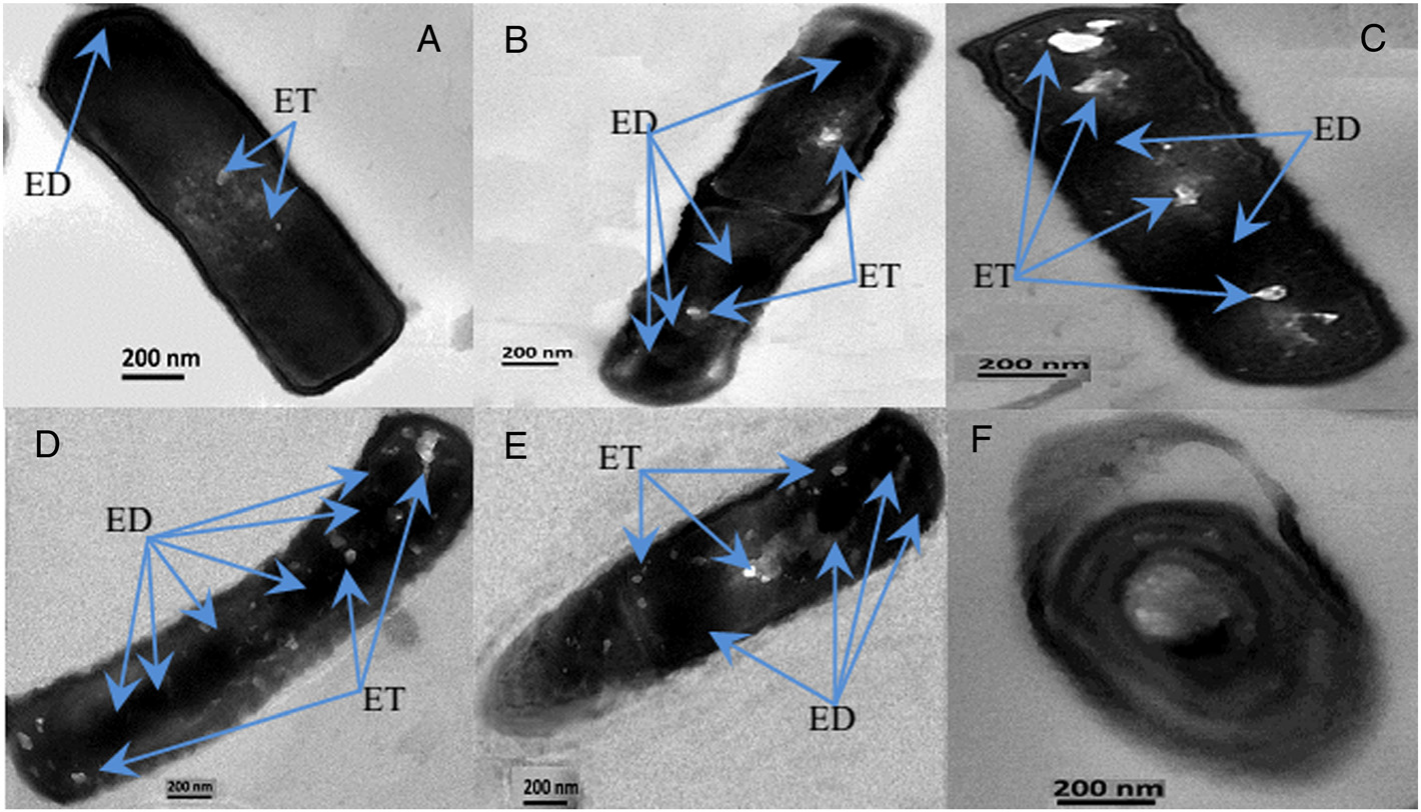
**TEM micrographs of cells of endophyte HB1310 sampled at 12 (A), 24 (B), 48 (C), 72 (D), and 96 h (E) (ED represents electron-dense, ET represents electron-transparent).** **(F)** TEM micrographs of endospore of endophyte HB1310 formed at later stage.

As early as 1996, Alvarez *et al.* studied the ultrastructure of the *R. opacus* strain PD630 using electron microscopy, and found that the bacterial cells contained two types of inclusion bodies: one being electron-dense (ED), and the other type being electron-transparent (ET) [28]. In this study, both types of inclusion bodies were observed in the cells of endophyte HB1310. The cell morphology changed significantly between the samples taken at 12 and 96 h (Figure 6A-E). After 12 h of cultivation, several ED and flat-to-spherical structures were observed close to the cytoplasmic membrane, while two fuzzy ET structures were also found, indicating preliminary formation of lipid inclusions (Figure 6A). When the cells had been incubated for 24 h, both ED and ET structures could be clearly observed, indicating that lipid inclusion formation started in full at this time (Figure 6B). In the sample withdrawn after 48 h of fermentation, the ED structures were enlarged and formed a nearly opaque circular shape that is clearly visible in the micrograph. Meanwhile, massive ET inclusions were formed, and some of these inclusions agglomerated to form large transparent domains (Figure 6C). Upon incubation between 72 and 96 h, most of the ED inclusions became detached from the cytoplasmic membrane and migrated into the cytoplasm, forming an opaque, roughly circular structure. The ET inclusions, by contrast, formed small circular or elliptic bodies and were distributed throughout the cytoplasm (Figure 6D,E). In addition, endospores are clearly observable at this stage (Figure 6F), which contained very little lipid content. As such, it is easily explainable that the bacterial lipid content was lower at 72 and 96 h than at 48 h, even though the nutritive cells showed high proportions of lipid inclusions at both sampling times. Therefore, in order to obtain a high lipid content, it is better to culture endophyte HB1310 for 48 h when using cotton stalk hydrolysate as the substrate.

Several bacteria have been reported to be able to produce cellular fatty acids, such as genera of *Streptomyces*, *Nocardia*, *Rhodococcus*, *Mycobacterium*, *Dietzia*, *Gordonia*, *Acinetobacter baylyi*, and *Alcanivorax borkumensis* [8]. The *R. opacus* strain PD630 is the oleaginous bacterium that has been studied the most, and has been previously reported to be capable of accumulating triacylglycerols (TAGs) accounting for up to 87% of the CDW when grown using a defined medium containing olive oil [28]. However, lower lipid contents were accumulated by this strain without an olive oil-containing growth medium. When sugar beet molasses and sucrose were used as substrates, *R. opacus* PD630 achieved lipid contents of 52% and 38.4% at the 30-L and 500-L scales [29]. *R. opacus* PD630 accumulated a maximum lipid content of 38% after about 144 h of cultivation in a medium with a high concentration of glucose [30]. However, this strain was not able to utilize xylose inherently. Its xylose metabolism was engineered to enable its use in lignocellulosic fuel production, such that the engineered strain could utilize xylose and glucose simultaneously for lipid production [17]. By contrast, the endophyte HB1310 can inherently utilize xylose and glucose simultaneously, and is also able to convert cotton stalk hydrolysate into cellular lipids. This strain also has the advantages of a high rate of cell growth and lipid accumulation, accumulating a maximum lipid content of 39.8% (w/w) at a culture time of 48 h.

### Comparison of the fatty acid profile of bacterial lipids with lipids of thin-shelled walnut and other microbial lipids

The composition of the fatty acids produced by endophyte HB1310 was analyzed. Proportional differences but no major variations in speciation were found in the samples taken from 12 to 96 h of culture time. The bacterial lipids produced after 48 h of culturing by endophyte HB1310 were compared with the lipids extracted from the fruits of the thin-shelled walnut. The main constituents of the bacterial lipids were identical to those of the thin-shelled walnut, indicating that endophyte HB1310 produces basically the same suite of lipids as its host, the thin-shelled walnut (Table 3), which may explain the ability of this strain to effectively produce lipids in the first place. The major fatty acids of both the bacterial and thin-shelled walnut lipids were found to be palmitic (C16:0), oleic (C18:1), and linoleic (C18:2) acids (Table 3). Although the major constituents were identical, there were again some proportional differences found. For example, the percentages of myristic (C14:0) and stearic (C18:0) acids in the bacterial lipids were higher than those in the lipids of the thin-shelled walnut. Some minor differences in composition were also discovered. Heptadecanoic (C17:0) acid was only found in bacterial lipid samples. Moreover, the lipids of the thin-shelled walnut contained numerous polyunsaturated fatty acids (PUFA) such as hexadecatrienoic (C16:3), eicosadienoic (C20:2), eicosatrienoic (C20:3), and docosenoic (C22:1) acids, which were not found in the bacterial lipids of endophyte HB1310, and although a significant amount of linolenic (C18:3) acid (11.8%) was detected in the lipids of the thin-shelled walnut, this compound represented only a small percentage (0.5%) of the composition of the bacterial lipids. A suitable composition of fatty acids for biodiesel production has been previously reported to contain mainly palmitic (16:0), stearic (18:0), oleic (18:1), linoleic (18:2), and linolenic (18:3) acids [31,32]. The bacterial lipid of endophyte HB1310 contains all of these compounds, with especially significant amounts of palmitic (16:0) and linoleic (18:2) acids. As such, this bacterium can be regarded as an oleaginous strain with high potential to be used as a feedstock for biodiesel production. The cetane number of fatty acid methyl esters, which is often used as an important metric for the evaluation of biodiesel composition, was estimated using the equation developed by Klopfenstein [33] and reference values summarized by Knothe *et al.* (2008) [34]. The cetane number of the bacterial lipids was estimated to be approximately 61.8, in good agreement with values obtained for other oleaginous microbes [31,32,35]. In addition, HB1310 possessed the characteristics of high palmitic (16:0) acid content like other oleaginous bacteria, yeasts, and microalgae (Table 3). However, it produced a high content of linoleic (18:2) acid, which was different from that in oleaginous bacteria and yeasts, but similar to that in thin-shelled walnut and microalgae, so this strain could be considered as an oleaginous bacterium producing a particular composition of fatty acid.

**Table 3:** **Comparison of the fatty acid profile of bacterial lipids with lipids of thin-shelled walnut and other microbial lipids**

**Organism**	**Substrate**	**Compositions of fatty acids (%, w/w)**							**Estimated cetane number**	**Reference**
		**C14:0**	**C16:0**	**C18:0**	**C16:1**	**C18:1**	**C18:2**	**C18:3**		
Thin-shelled walnut	-	0.15	12.48	0.27	0.75	21.54	36.48	11.84	-	This study
HB1310	Cotton stalk hydrolysate	5.28	28.33	3.83	8.6	8.59	32.13	0.5	61.8	This study
*Rhodococcus opacus* PD630	Glucose	2.0	27.8	4.1	10.7	20.7	nd	nd	-	Kurosawa *et al.*, 2010 [30]
*Rhodococcus opacus* PD630	1.0% (w/v) molasses	5.1	31.0	18.9	10.8	18.9	nd	nd	-	Voss and Steinbüchel, 2001 [29]
*Lipomyces starkeyi*	Glucose	0.7	36.3	5.0	4.2	50.1	3.7	nd	61.1	Lin *et al.*, 2011 [26]
*Lipomyces starkeyi*	Glucose and xylose	0.4	33.9	7.5	3.4	50.6	3.9	nd	61.0	Tapia *et al.*, 2012 [35]
*Lipomyces starkeyi*	Hemicellulose hydrolysate	nd	30.3	12.6	0.7	46.7	7.0	nd	62.9	Anschau *et al.*, 2014 [18]
*Micractinium reisseri*	Wastewater	2.07	28.75	5.51	0.71	10.85	24.44	26.75	-	Abou-Shanab *et al.*, 2014 [36]

nd represents “not determined”.

## Conclusions

The present investigation is the first report of an oleaginous endophytic bacterium isolated from the thin-shelled walnut, and has demonstrated that the isolated strain, endophyte HB1310, is capable of producing high lipid contents using cotton stalk hydrolysate as a substrate. Satisfactory productivities were obtained after 48 h of batch fermentation in a bioreactor, with a maximum lipid productivity of 2.3 g/L, lipid content of 39.8% (w/w), and cell dry weight (CDW) of 5.7 g/L. These results suggest that endophyte HB1310 can rapidly produce high lipid contents. The fatty acid profile of the bacterial lipids indicates that this strain has the potential to be used as a feedstock for the production of biodiesel.

## Methods

### Preparation of sterilized kernels of thin-shelled walnut and cotton stalk hydrolysate

Mature thin-shelled walnuts (variety *Akesu* 185) were harvested in early October of 2012 from an orchard in Xinjiang Akesu, China. The fruits were shelled, and the kernels removed. The kernels were then dipped in Tween80 for 15 min before being subjected to treatment with a peelremover and sterilization of the surface. The surface was sterilized using the following steps: treatment with a 75% ethanol solution for 5 min, followed by three to four washes with sterile water, treatment with a 1% H_2_O_2_ solution for 2 min, another three to four washes with sterile water, a second treatment with 75% ethanol solution for 1 min, and a final three to four washes with sterile water. A 0.1-mL sample from the water used for the last washing was taken and spread on an LB plate to test the sterility. The sterilized kernels were dried on sterile filter paper, and ground into powder with a sterile mortar to enable the isolation of endophytes.

Cotton stalks (of variety *Gossypiumhirsutum Linn*Zhong 35) were harvested in early November of 2012 from a cotton field in Xinjiang Alaer, China. The stalks were air-dried, ground into fragments, and sifted using a 20-mesh sieve before being hydrolyzed. The cotton stalk was originally composed of cellulose 38.15% (w/w), lignin 18.35% (w/w), and hemicellulose 12.91% (w/w). Hydrolysis was performed at 121°C for 30 min using a 4% (v/v) sulfuric acid solution and a solid-liquid ratio of 1:5, these being the optimum hydrolysis conditions as reported previously by our group [22]. After hydrolysis, the mixture was separated by filtration and centrifugation at 4,000 rpm. The composition of the raw hydrolysate obtained was glucose 31.91 g/L, xylose 11.32 g/L, arabinose 1.80 g/L, acetic acid 1.56 g/L, hydroxymethylfurfural (HMF) 0.04 g/L, furfural 0.01 g/L, fatty acid compounds (mainly composed of cyclopentanecarboxylic acid, pentadecyl acid, tetradecyl acid, heptadecanoic acid) 0.008 g/L, and aromatic compounds (mainly composed of naphthalene 2,6-dicarboxylic acid, phthalic acid, 3-phenanthrol, 9-(o-propylphenyl) nonanoic acid, 11-dimethyl-1,2,3,4-tetrahydro-1,4-methanophenazine-1-carboxylic acid, 2-phenyl dioxolane, 7-(o-pentylphenyl) heptanoic acid) 0.02 g/L. The last four components made the solution toxic with dark color, which inhibited growth of the endophyte, so the hydrolysate was then detoxified and decolorized using a calcium hydroxide solution and macroporous resin LS610, respectively, as previously reported, resulting in a product of which the main reducing sugar components were glucose and xylose [21,22], and other components were barely detected. Finally, the concentration of reducing sugars in cotton stalk hydrolysate was concentrated to 50 g/L before use as the medium in the fermentation experiments.

### Media

Three types of media were used in this investigation: 1) isolation media: the Luria-Bertani (LB) medium consisted of peptone (10 g/L), yeast extract (5 g/L), NaCl (10 g/L), and agar (22 g/L) in water, with an initial pH of 7.0; the water-agar (WA) medium was a 22 g/L solution of agar in 1,000 mL of water, and the initial pH was 6.5-7.0; designed isolation medium #1 (DI1) was prepared using D-xylose (5 g/L), glucose (5 g/L), yeast extract (5 g/L), peptone (2.5 g/L), NaCl (5 g/L), and agar (22 g/L) in aqueous solution, with an initial pH of 7.0; designed isolation medium #2 (DI2) used a base of cotton stalk hydrolysate with a reducing sugar concentration of 50 g/L, to which was added yeast extract (5 g/L), peptone (5 g/L), and agar (22 g/L), this solution also had an initial pH of 7.0. 2) Seed medium: the liquid medium of DI1 was used as the seed medium. 3) Fermentation medium: the fermentation medium was made by adding KH_2_PO_4_ (0.5 g/L) and MgSO_4_ · 7H_2_O (1 g/L) to 1,000 mL of cotton stalk hydrolysate; the initial concentration of reducing sugar was adjusted to 50 g/L in all experiments, while varying C/N ratios were achieved by adding different concentrations of yeast extract.

### Isolation of oleaginous endophyte

Sterilized and powdered thin-shelled walnut kernels were inoculated on an agar plate of LB, WA, and DI1 media and cultured in a constant-temperature incubator at 37 ± 2°C for three to five days. The growing clones were inoculated onto a DI2 plate for rescreening for endophytes which were able to utilize the reducing sugars of cotton stalk hydrolysate. The candidate bacteria were isolated from the DI2 plate, purified using a dilution-plate method with repeated streaking, and preserved in DI1 for subsequent fermentation. A control plate with cotton stalk hydrolysate removed from DI2 was also used to verify the candidate bacteria’s preference for using cotton stalk hydrolysate as a carbon source.

### Identification of oleaginous endophyte and physiological and biochemical properties

Genomic DNA was extracted from endophytic cells in the logarithmic growth phase using an Ezup Column Bacteria Genomic DNA Purification Kit (Sangon, China) according to the manufacturer’s instructions. The 16S rDNA gene was amplified by PCR using the primer pair 27 F/1492R. The PCR products were sequenced, and the 16S rDNA sequence was aligned and identified by GenBank using the BLAST program.

Gram staining of the bacterium was performed with the Hucker method, which was described in detail by Doetsch [37]. The morphology of the bacterium was examined using an optical microscope DM1000 LED (Leica, Germany). Examinations of bacterial physiological and biochemical properties were performed according to the protocols described in the *Identification Manual of Systematic Bacteriology* [38]. The carbon source and nitrogen source utilization experiments and the temperature and pH experiments were carried out to preliminarily evaluate the experimental conditions for growth and lipid production of the bacterium. The carbon source and nitrogen source utilization experiments were developed using basal medium described in the *Identification Manual of Systematic Bacteriology* [38], with culture temperature 37°C and pH7.0. The temperature and pH experiments were developed using DI2 medium. In the temperature experiments, the pH value was modulated to 7.0, and in the pH experiments, the culture temperature was 37°C. All of the experiments were performed in triplicate.

### Fermentation of cotton stalk hydrolysate

The isolated endophyte was used as an inoculum for the fermentation of cotton stalk hydrolysate. A 250-mL Erlenmeyer flask containing 100 mL of seed medium was inoculated with three loopfuls of cells taken from a 24-hour-old activation slant and incubated at 37°C on a rotary shaker at 150 rpm for 16 to 20 hours. Seed strains with OD_600_ = 0.8-1.0 were inoculated into the fermentation medium described above in a proportion of 10% (v/v). For each fermentation sample, 100 mL of fermentation medium was loaded into a 500-mL Erlenmeyer flask and incubated in a constant-temperature vibrator shaking at 120 rpm. At culture times of 12, 24, 48, 72, and 96 h, five flasks of cultures were withdrawn: two were used for the determination of strain biomass and three for lipid extraction and analysis of the reducing sugars. In the shake-flask experiments, for evaluating the effects of different C/N ratios on lipid production and bacterial growth, the initial concentration of reducing sugar was adjusted to 50 g/L, and the C/N ratios were adjusted to 25/1, 50/1, 75/1, 100/1, 125/1, 150/1 with culture temperature 37°C and pH7.0; for evaluating the effects of three temperature (30, 37, 45°C) and two pH values (6.5, 7.0) on lipid production and bacterial growth, the temperature experiment was developed with C/N ratio 50 and pH7.0, and the pH experiment was developed with C/N ratio 50 and temperature 30°C. In the scale-up experiment, seed inoculums were inoculated into a 10-L bioreactor (BioFlo 310, Eastbio, China) for batch fermentation using the optimum conditions determined in the shake-flask experiments. Both the shake-flask experiments and the scale-up experiment in the bioreactor were performed in triplicate. All of the data were displayed using a descriptive statistical method, and the data in the C/N ratio experiments were also analyzed with the Tukey test.

### Fluorescence microscopy and transmission electron microscopy (TEM)

Bacterial cell suspensions (1000 μL) were harvested by centrifugation at 12,000 rpm for 5 min, and stained using Nile red solution (0.5 μg/mL) according to the method described by Wältermann *et al.* [27]. The stained cells were then observed and photographed with an Axio Image A1 fluorescence microscope (Ceiss, Germany).

Transmission electron microscopy (TEM) samples were prepared using the follow procedure: cells were first fixed with 2.5% (w/v) glutaraldehyde overnight, followed by three washes lasting 20 min each with 0.1 M PBS (pH =7.2), a second fixing step with 1% (w/v) osmium tetroxide for 120 min, and three washes of 15 min each with 0.1 M PBS (pH =7.2). The water was then removed using a graded water-ethanol series (30%, 50%, 70%, 85%, 95% (v/v) ethanol) in which samples were exposed to each solution for 10 min, and dehydrated three times (also for 10 min each repetition) using 100% (v/v) ethanol. The samples were permeated with a 1:1 mix of ethanol and LR White resin for 2 h, and embedded in an embedding plate. The resin was polymerized at 70°C for 24 h. 200-nm-thick sections were cut using an Ultracut Microtome (Leica EM UC7, Germany) and stained with uranyl acetate/lead citrate. Finally, the sections were mounted on 200-mesh grids and examined using a Jel-1400 microscope (Japan).

### Extraction of lipids from thin-shelled walnut fruit

Peeled walnut kernels were dried and ground, and then extracted by hexane at a solid-liquid ratio of 1:5 for 1 h. The extract was filtered and the liquor was collected, with this step being repeated according to the methods reported by Mao and Hua [16]. The extracts were evaporated with a rotary evaporator for removal of hexane. The extracted oil left in the rotating bottle consisted of thin-shelled walnut lipids, which were then converted into fatty acid methylesters (FAME) and analyzed using GC/MS gas chromatography (TRACE DSQ, USA), as described below.

### Measurement of bacterial biomass, fatty acids, and reducing sugars

Five flasks of cultures were withdrawn at various times during bacterial fermentation: two samples from each time point were used for the determination of strain biomass, and three samples were used for lipid extraction and reducing sugar analysis. All samples were centrifuged at 4,000 rpm for 20 min before use.

The supernatant of the two flasks used for mass determination was then discarded and the strain mud was collected and washed twice with normal saline. The washed mud was then dried in an electro-thermostatic drier at 80°C until a constant weight was obtained, which was defined as the cell dry weight (CDW).

The supernatant of the remaining three flasks was filtered through a 0.22-μmmillipore filter for HPLC determination of reducing sugar concentrations. The strain mud was washed twice with distilled water and treated with 4 M hydrochloric acid before incubation at 80°C for 1 h, followed by cooling to 4°C for 15 min. The lipids were then extracted and quantified using the Bligh-Dyer method [39], while the total lipid content was measured gravimetrically. Cellular lipids were converted to FAME using a previously reported method [40], and then analyzed using a GC/MS gas chromatography system (TRACE DSQ, USA) with a DB-5MS capillary column (of dimensions 30 m × 0.25 mm × 0.25 μm). The temperature program used for the analysis of the FAME samples was as follows: the initial temperature, 70°C, was held for 5 min, after which the temperature was gradually increased at a rate of 25°C per minute to 210°C, where it was held for 1 min before increasing to 240°C at 2.4°C per minute, and then to a maximum of 270°C at 5°C per minute. The maximum temperature was held for 15 min. Helium was used as the carrier gas with a constant flow rate of 1 mL · min^-1^. The injection volume was 1 μL.

The total concentration of reducing sugars in the hydrolysate was determined by the 3,5-dinitryl-salicylic acid reagent (DNS) method reported by van Soest *et al.* [41]. Glucose and xylose concentrations were detected using a high-performance liquid chromatograph (HPLC) (Shimadzu LC-2A) equipped with a refractive index detector. A Cosmosil NH_2_ column (5 μm, 4.6 mm × 250 mm) was used with a solution of acetonitrile and water (75:25) as the eluent. Tests were conducted using an eluent flow rate of 1.0 mL/min at a temperature of 40°C, and an injection volume of 20 μL. The glucose and xylose concentrations were calculated based on calibration curves that were built using glucose and xylose standards using the same detecting conditions as above.

### Calculation of bacterial lipid production properties

Several indices were used to describe the efficiency of bacterial lipid production. Namely, the lipid content (C_L_) and lipid yield produced from sugars (Y_L/S_) were defined and calculated as follows:

1 $$ {\mathrm{C}}_{\mathrm{L}}\left(\%\right)=\left(\mathrm{D}{\mathrm{W}}_{\mathrm{L}}/\mathrm{D}{\mathrm{W}}_{\mathrm{B}}\right)\times 100 $$

2 $$ {\mathrm{Y}}_{\mathrm{L}/\mathrm{S}}\left(\mathrm{g}/\mathrm{g}\right) = \mathrm{D}{\mathrm{W}}_{\mathrm{L}}/\left({\mathrm{W}}_{\mathrm{Si}}\hbox{-} {\mathrm{W}}_{\mathrm{Sf}}\right) $$

where C_L_ = lipid content (%), DW_L_ = dry weight of lipid (g), DW_B_ = dry weight of cell biomass (g), Y_L/S_ = lipid production from sugars (g/g), W_Si_ = initial sugars weight (g), W_Sf_ = final sugars weight (g).

## Abbreviations

CDW: cell dry weight
C/N: carbon/nitrogen
DNS: 3,5-dinitryl-salicylic acid reagent
FAME: fatty acid methyl ester
GC-MS: gas chromatography-mass spectrometry
HPLC: high performance liquid chromatography
OD: optical density
PBS: phosphate-buffered saline
TEM: transmission electron microscopy
Y_L/S_: lipid yield produced from sugars

## References

[1] Nigam PS, Singh A (2011). Production of liquid biofuels from renewable resources. Prog Energy Combust Sci.

[2] Wahlen BD, Morgan MR, McCurdy AT, Willis RM, Morgan MD, Dye DJ, Bugbee B, Wood BD, Seefeldt LC (2013). Biodiesel from microalgae, yeast, and bacteria: engine performance and exhaust emissions. Energ Fuels.

[3] Hetzler S, Steinbuchel A (2013). Establishment of cellobiose utilization for lipid production in *Rhodococcus opacus*PD630. Appl Environ Microbiol.

[4] Ratledge C (1991). Microorganisms for lipids. Acta Biotechnol.

[5] Azocar L, Ciudad G, Heipieper HJ, Navia R (2010). Biotechnological processes for biodiesel production using alternative oils. Appl Microbiol Biotechnol.

[6] Alvarez HM, Steinbüchel A (2002). Triacylglycerols in prokaryotic microorganisms. Appl Microbiol Biotechnol.

[7] Meng X, Yang J, Xu X, Zhang L, Nie Q, Xian M (2009). Biodiesel production from oleaginous microorganisms. Renew Energ.

[8] Brigham CJ, Kurosawa K, Rha C, Sinskey AJ (2011). Bacterial carbon storage to value added products. Microb Biochem Technol.

[9] Patnayak S, Sree A (2005). Screening of bacterial associates of marine sponges for single cell oil and PUFA. Lett Appl Microbiol.

[10] Xu ZH, Gao DM, Song XL, Xu Y (2012). A review of endophyte and its use and function. 2012 International Conference on Environmental Engineering and Technology Advances in Biomedical Engineering.

[11] Castillo UF, Strobel GA, Ford EJ, Hess WM, Porter H, Jensen JB, Albert H, Robison R, Condron MA, Teplow DB, Stevens D, Yaver D (2002). Munumbicins, wide-spectrum antibiotics produced by *Streptomyces* NRRL 30562, endophytic on *Kennedia nigriscans*. Microb.

[12] Strobel GA (2002). Rainforest endophytes and bioactive products. Crit Rev Biotechnol.

[13] Peng XW, Chen HZ (2007). Microbial oil accumulation and cellulase secretion of the endophytic fungi from oleaginous plants. Ann Microbiol.

[14] Strobel GA, Knighton B, Kluck K, Ren Y, Livinghouse T, Griffin M, Spakowicz D, Sears J (2008). The production of myco-diesel hydrocarbons and their derivatives by the endophytic fungus *Gliocladium roseum*(NRRL 50072). Microbiol.

[15] Dey P, Banerjee J, Maiti MK (2011). Comparative lipid profiling of two endophytic fungal isolates – *Colletotrichum* sp. and *Alternaria* sp. having potential utilities as biodiesel feedstock. Bioresour Technol.

[16] Mao XY, Hua YF (2011). Research of chemical composition and properties of thin-shelled walnut in Xinjiang. Sci Technol Food Ind.

[17] Kurosawa K, Wewetze SJ, Sinskey AJ (2013). Engineering xylose metabolism in triacylglycerol producing *Rhodococcus opacus* for lignocellulosic fuel production. Biotechnol Biofuels.

[18] Anschau A, Xavier MC, Hernalsteens S, Franco TT (2014). Effect of feeding strategies on lipid production by *Lipomyces starkeyi*. Bioresour Technol.

[19] Liu W, Wang Y, Yu Z, Bao J (2012). Simultaneous saccharification and microbial lipid fermentation of corn stover by oleaginous yeast *Trichosporon cutaneum*. Bioresour Technol.

[20] Liang YN, Tang TY, Umagiliyage AL, Siddaramu T, McCarroll M, Choudhary R (2012). Utilization of sorghum bagasse hydrolysates for producing microbial lipids. Appl Energ.

[21] Zhang Q, Li Y, Xia L, Liu Z, Pu Y (2014). Enhanced xylitol production from statistically optimized fermentation of cotton stalk hydrolysate by immobilized *Candida tropicalis*. Chem Biochem Eng Q.

[22] Zhang Q, Li YB, Li JJ, Ma CM: **Dilute acid hydrolysis of cotton stalks and ethanol production from hydrolytic liquids.** In *Proceedings 2011 International Conference on Materials for Renewable Energy & Environment: 10-12 May, 2011.*ᅟ Edited by Ni WD. Shanghai: 2011:459–463.

[23] Ratledge C, Wynn JP (2002). The biochemistry and molecular biology of lipid accumulation in oleaginous microorganisms. Adv Appl Microbiol.

[24] Ageitos JM, Vallejo JA, Veiga-Crespo P, Villa TG (2011). Oily yeasts as oleaginous cell factories. Appl Microbiol Biotechnol.

[25] Zhang Z, Zhang X, Tan T (2014). Lipid and carotenoid production by *Rhodotorula glutinis* under irradiation/high-temperature and dark/low-temperature cultivation. Bioresour Technol.

[26] Lin J, Shen H, Tan H, Zhao X, Wu S, Hu C, Zhao ZK (2011). Lipid production by *Lipomyces starkeyi* cells in glucose solution without auxiliary nutrients. J Biotechnol.

[27] Wältermann M, Hinz A, Robenek H, Troyer D, Reichelt R, Malkus U, Galla HJ, Kalscheuer R, Stöveken T, von Landenberg P, Steinbüche A (2005). Mechanism of lipid-body formation in prokaryotes: how bacteria fatten up. Mol Microbiol.

[28] Alvarez HM, Mayer F, Fabritius D, Steinbüchel A (1996). Formation of intracytoplasmic lipid inclusions by *Rhodococcus opacus* strain PD630. Arch Microbiol.

[29] Voss I, Steinbüchel A (2001). High cell density cultivation of *Rhodococcus opacus* for lipid production at a pilot-plant scale. Appl Microbiol Biotechnol.

[30] Kurosawa K, Boccazzi P, de Almeida NM, Sinskey AJ (2010). High-cell-density batch fermentation of *Rhodococcus opacus*PD630 using a high glucose concentration for triacylglycerol production. J Biotechnol.

[31] Knothe G (2009). Improving biodiesel fuel properties by modifying fatty ester composition. Energ Environ Sci.

[32] Tanimura A, Takashima M, Sugita T, Endoh R, Kikukawa M, Yamaguchi S, Sakuradani E, Ogawa J, Shima J (2014). Selection of oleaginous yeasts with high lipid productivity for practical biodiesel production. Bioresour Technol.

[33] Klopfenstein WE (1985). Effect of molecular weights of fatty acid esters on cetane numbers as diesel fuels. J Am Oil Chem Soc.

[34] Knothe G (2008). “Designer” biodiesel: optimizing fatty ester composition to improve fuel properties. Energ Fuel.

[35] Tapia EV, Anschau A, Coradini AL, Franco TT, Deckmann AC (2012). Optimization of lipid production by the oleaginous yeast *Lipomyces starkeyi*by random mutagenesis coupled to cerulenin screening. AMB Express.

[36] Abou-Shanab RAI, El-Dalatony MM, EL-Sheekh MM, Ji MK, Salama ES, Kabra AN, Jeon BH (2014). Cultivation of a new microalga, *Micractinium reisseri*, in municipal wastewater for nutrient removal, biomass, lipid, and fatty acid production. Biotechnol Bioproc E.

[37] Doetsch RN, Gerhardt P, Murray RGE, Costilow RN, Nester EW, Wood WA, Krieg NR (1981). Determinative methods of light microscopy. Manual of Methods for General Bacteriology.

[38] Dong XZ, Cai MY (2001). Identification Manual of Systematic Bacteriology.

[39] Bligh EG, Dyer WJ (1959). A rapid method of total lipid extraction and purification. Can J Biochem Phys.

[40] Lewis T, Nichols PD, McMeekin TA (2000). Evaluation of extraction methods for recovery of fatty acids from lipid-producing microheterotrophs. J Microbiol Meth.

[41] van Soest PJ, Robertson JB, Lewis BA (1991). Methods for dietary fiber, neutral detergent fiber, and non-starch polysaccharides in relation to animal nutrition. J Dairy Sci.

